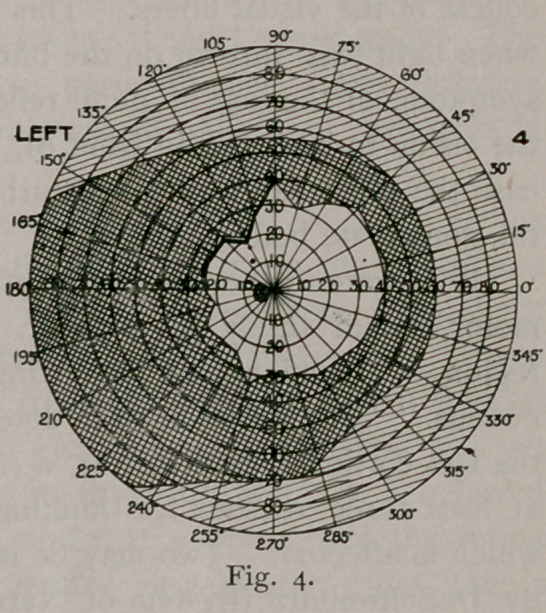# Hemianopsia1Read at the thirty-first annual meeting of the Medical Association of Central New York, at Auburn, October 18, 1898.

**Published:** 1899-02

**Authors:** F. W. Higgins

**Affiliations:** Cortland, N. Y.


### HEMIANOPSIA.[1]

By F. W. HIGGINS, M. D., Cortland, N. Y.

I HAVE recently been quite interested in a case of hemianopsia, which developed under my observation, and should like to bring to your notice some features of the subject which seem to me of importance.

As general practitioners, we are too apt to relegate any symptom connected with vision to the eye men, or to ignore it altogether. Max Knies has written a very valuable little book on the relationship of the eye and its diseases to the general diseases of the body. The case which I shall attempt to detail is one of that class which cannot be neglected by the man in general practice any more than by the pure specialist. But it seems to me of more practical importance to the former class. He who treats the eye alone will rarely have an opportunity to discover such cases. If they are not recognised by the attending physician they may easily be overlooked altogether. The treatment is clearly within the field of internal medicine.

Case I.—Widow, aged sixty. Thirteen months before the present attack she developed left-sided hemiplegia, from which the leg has partially recovered, but the arm remains useless. I have frequently seen her since that time, and was sent for August 30, 1898. She was sitting in her chair as usual, complained of some ill-defined feelings of depression, but I obtained no definite symptom until she mentioned that there was a confusion in vision. Further quizzing developed the fact that objects at her left looked blurred. Then I found that if she fixed her attention with her right eye upon my face, any movement of my right hand looked foggy and indistinct. At this time the left eye was only slightly affected. With this she could see my left hand distinctly. Her daughter had remarked for two days that she had not cared to read and was not quite so well. She could see better with right eye covered. Four days later the left field was quite obliterated in the right eye, and limited to hand movements in the left.

Testing her vision with a newspaper developed a peculiar state of affairs. She could read the words on the ends of the lines in a column, but not the first half, so that she could make no sense of an article. In the larger type of the advertisements she could see the last letter of a word, but not the first. Looking at the word PALMER in large capitals she could see the letters E R only with the right eye, and M E R with the left.

---

1. Read at the thirty-first annual meeting of the Medical Association of Central New York, at Auburn, October 18, 1898.

It is rather difficult for me to understand why her eye could not be directed a little further to the left, so that in some manner the first half of the word could be caught, but she was certainly unable to do so. In the central scotomata of choroiditis the patient learns to get along very well by slightly altering the visual axis and so manages to see the whole of an object. It seems to me possible that a young person with hemianopsia would acquire the same trick and so apparently increase the field of vision.

The accurate mapping out of the visual field by a perimeter was first done September 15th, sixteen or eighteen days after the lesion occurred. I was surprised to find so regular a hemianopsia in a case which was discovered almost by accident. Noyes, Starr and others have warned us that many cases of half blindness are overlooked by

Fig. 1.

Fig. 2.

physician and patient. My experience with this case shows me how easily this might occur. Even intelligent patients are apt to complain only of a confusion in vision, or at most that the distance is worse on one side, which they explain by the eye on that side being most affected. At the time these fields were taken (Figs. 1 and 2) she could not distinguish strong light reflected upon the temporal half of the right retina, but could upon the nasal half of the left. The left half of the field in the right eye was an absolute blank, but in the left eye it seemed to her as if a thick fog covered everything and she could only distinguish light from darkness. Since that time there has been a gradual improvement.

The fields taken October 7th (Figures 3 and 4) show decided enlargement of the fields to the left, although the limitation of the left field is still plain. Wilbrand claims that six weeks must elapse before the fields of vision can be utilised in localisation, since before that time distant lesions will reflect their influence.

We have here clear evidence of a cerebral lesion, causing hemianopsia. It began about August 28th, was marked in the right eye August 30th and complete September 3d. On September 9th she could distinguish light again, and, since, there was a gradual improvement until she could distinguish movements. The left eye went through the same stages, but the blindness was not so complete. On September 28th she could read a whole line of ordinary type with the left eye, and a coarse line slowly and with difficulty with the right. There is now no blur in the left eye, but the right confuses her and she can see better with it closed. She is now able to read a newspaper again slowly, and I expect she will not realise the

Fig. 3.

Fig. 4.

hemianopsia, although the left fields of vision, if tested, will remain somewhat narrowed. It is fortunate for her that the paretic field is on the left side. Noyes remarks that loss of the right field is most troublesome, as the eye cannot then anticipate the coming words.

The parallel to the attack of hemiplegia is close. This required about one week to reach its climax, and since, there has been a gradual improvement, but not a complete recovery. Much of the time the scotomata have been positive—a point favorable in prognosis.

It is interesting to attempt to locate the lesion which occurred. We should know in what part of the brain the focus lies for prognosis, for treatment, and it might be for surgical intervention.

So late as 1884, so good an authority as Starr, at the close of a most interesting article on the subject, stated that hemiopia as a symptom had no localising value. The mass of observation, including his own, since that date has so extended our knowledge that he would hardly make this statement today. It may be that aphasia and motor paralyses can give us more definite indications, but much satisfaction is possible in the study of a case of hemianopsia. It is even possible that surgical exploration would be warranted on the date obtainable.

The attempt at localisation in our case would result about as follows. Since both eyes are affected we must evidently look behind the chiasm. Yet left homonomous hemianopsia may occur from a lesion in the left optic tract, thalmus opticus, geniculate bodies, internal capsule, the radiating fibers of Gratiolet, or the gray matter of some portion of the occipital region.

One cardinal sign at once eliminated the anterior half of this long course of the visual fibers. This was the retention of the pupil reflex when light was thrown on the blind half of the retina. Wernicke's symptom, or the loss of pupil reflex from the blind side, would show the lesion to be in, or in front of, the primary optic centers. In my case the reflex arc was not disturbed, so the lesion must be beyond the geniculate bodies.

If it were in the internal capsule, other symptoms, sensory and motor, would surely be present. We are shut up to the occipital region. "Hemianopsia occurring alone points to lesion of the cuneus." Hence we have to determine only between the cortex and the sub-cortical white matter, *i. e.*, the radiating fibers. There are at least three reasons for thinking that in this case it is the cortex which is affected. Two may be inferred from categorical statements by DeSchweinitz (System of Nervous Diseases, Dercum., p. 772.) " If the hemianopsia is *relative* the lesion must be in the cortex. Elsewhere it produces *absolute* hemianopia. However, cortical lesions are not excluded by absolute hemianopsia." My case, it will be remembered, was at one time relative in one eye, absolute in the other. " Contraction of the preserved half field is most common with lesions of the cortex, but it may also occur with lesions of the tract." The fields in my case were manifestly contracted.

The third reason is a statement of my patient that on the first day of the attack, for a few hours, she saw " crawly things " on what afterward became the blind side. This must have been due to irritation of the visual center. L. Putzel (*Medical Record*, June 2, 1898,) saw a case of long continued hallucinations on the blind side and found the lesion cortical. It appears that hallucinations can arise only from irritation of the gray matter. Thus, so far as these symptoms can be trusted, they would indicate that this lesion was cortical instead of subcortical.

There was no hesitancy in recognising the name or the use of an object that could be seen, so the region of the angular gyrus probably escaped. It seems fairly well settled that physical blindness resides near where Ferrier first located the center for vision. There was no want of balance, no dizziness, no aphasia, in fact no other symptom than the hemianopsia, save a slight malaise, so that the lesion is probably a small one and limited to the gray matter near the right cuneus.

It will be noticed that the boundary line of the hemianopic field is not a straight line, passing through the point of fixation. This classical condition is rarely found. Often the blindness falls a few degrees short of the meridian of the fovea ; it never oversteps it. The dividing line is not always straight or perpendicular. Any variation may occur, owing, as Wilbrand contends, to individual variations in the distribution of the nerve.

Then the marked prominence out into the blind field that serves to retain vision at the macula is noticeable. This is found in most cases. It is supposed that the macula is supplied from both hemispheres. It even retains vision in double hemianopsia. There is a theory that the fibers coming from the macula have a special distinct destination in the occipital gray matter, but no one has yet been fortunate enough to determine its exact location. It is believed that the field of vision is a mosaic, and that every part of the retina has a corresponding part of gray matter as a center. But Hun's case, published in 1887, so far as I know, remains unique in the finer localisation within the cuneus. He found that the lower part of the left field of vision was attended with atrophy of the lower part of the left cuneus.

Color sense is sometimes disturbed in a different way than the field for white. Color blindness has been produced by central lesions. The field for red in this case runs parallel to that for white.

We have all noticed the effort attending a motion in cases of paresis of central origin. Exhaustion soon follows any exertion, even when there is ability to perform the action. The same thing was very manifest in the expressions of my case of supposed occipital lesion. The retinal tire is also shown by the inside line in Figs. 2 and 3. The retina was becoming exhausted by the time the second est was made, so narrowing the field about ten degrees. In Fig.

4 the second line almost coincides with the first, showing improvement in the case.

One interesting feature is that vision was much poorer in the right eye than in the left. In the left the blind side was never so absolutely blind, and in the seeing field she could read finer type. She could see better with the right covered than with both together. Now the vision in the right eye is 6/30 minus, in the left, 6/12 plus and minus.

The reason for this difference becomes clear by dipping a little into comparative anatomy. In birds the optic tracts at the chiasm cross entirely, so that the whole right eye is connected with the left brain. As we rise in the scale of animal life, the eyes come more and more to the front, so binocular vision is to some extent possible. Parallel to this a larger and larger bundle of the optic tract supplies the outer half of the retina of the eye on its own side. But even in man, in whom we consider the axes of vision to be parallel, a trace of his development remains in the crossed bundle being larger than the uncrossed in the proportion of three to two. So in occipital lesions, if the destruction is not complete, we find the greater disturbance in the eye on the opposite side.

## Figures and Tables

**Fig. I. f1:**
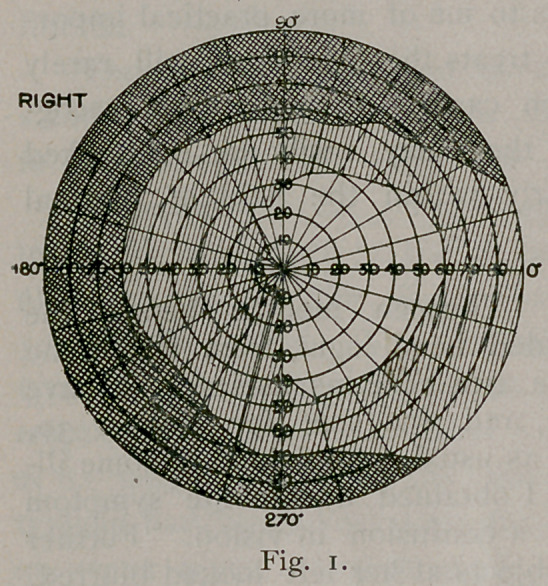


**Fig. 2. f2:**
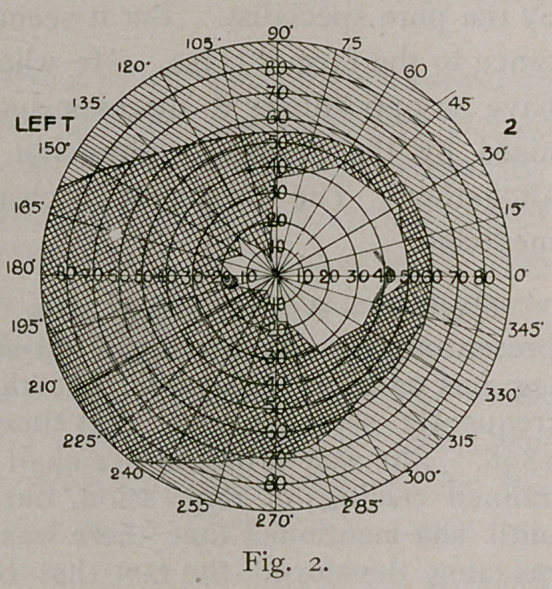


**Fig. 3. f3:**
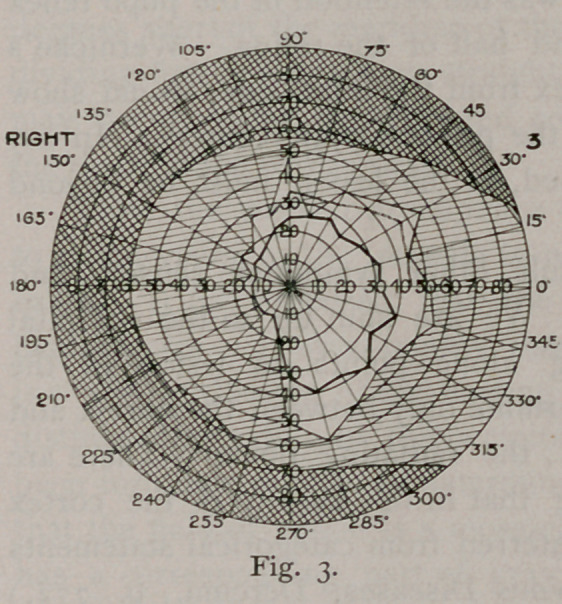


**Fig. 4. f4:**